# Photoacoustic and high-frequency ultrasound imaging of systemic sclerosis patients

**DOI:** 10.1186/s13075-020-02400-y

**Published:** 2021-01-12

**Authors:** Khalid Daoudi, Brigit E. Kersten, Cornelia H. M. van den Ende, Frank H. J. van den Hoogen, Madelon C. Vonk, Chris L. de Korte

**Affiliations:** 1 Medical UltraSound Imaging Center (MUSIC), Department of Radiology and Nuclear Medicine, Sint Maartenskliniek Post 766, PO box 9101, 6500 HB Nijmegen, The Netherlands; 2 Department of Rheumatic Diseaes Radboud University Medical Center, Sint Maartenskliniek Post 766, PO box 9101, 6500 HB Nijmegen, The Netherlands; 3 Department of Rheumatollogy, Sint Maartenskliniek Post 766, PO box 9101, 6500 HB Nijmegen, The Netherlands

**Keywords:** Systemic sclerosis, Early diagnosis, Photoacoustic, High-frequency ultrasound, Skin thickening

## Abstract

**Introduction:**

Systemic sclerosis starts with an early phase characterized by Raynaud’s phenomenon, puffy fingers/hands, autoantibodies, and a scleroderma nailfold microscopic pattern. Alterations in the nailfold microscopic pattern are not evident in all early SSc patients. Photoacoustics (PA) and high-frequency ultrasound (HFUS) could fulfill this need. The former can measure oxygen saturation while the latter can measure skin thickening. We hypothesize that photoacoustics and high-frequency ultrasound can distinguish (early) SSc patients from individuals with primary Raynaud’s phenomenon (PRP) by measuring oxygenation of the fingertip and skin thickening.

**Methods:**

We compared measurements of oxygenation and skin thickness of the third finger between (early) SSc patients and PRP individuals and healthy controls. The spearman rank correlation was used to analyze an association between capillary density and oxygen saturation of the fingers.

**Results:**

Thirty-one adult subjects participated in this study: twelve patients with SSc, 5 patients with early SSc, 5 volunteers with PR, and 9 healthy controls.

We found a significant difference in oxygen saturation between (early) SSc patients (80.8% ± 8.1 and 77.9% ± 10.5) and individuals with PRP (93.9% ± 1.1).

Measurements of skin thickening showed a significant difference in (early) SSc patients compared to individuals with PRP (0.48 ± 0.06 mm and 0.51 ± 0.16 mm vs. 0.27 ± 0.01 mm). There was no significant difference between healthy and PRP individuals in oxygenation or skin thickening.

**Conclusion:**

Photoacoustic and high-frequency ultrasound could help to distinguish between (early) SSc, PRP, and healthy individuals in both oxygenation and skin thickening.

## Introduction

Systemic sclerosis (SSc) is an autoimmune disease characterized by a triad of inflammation, vasculopathy, and fibrosis of the skin and internal organs such as the lungs and heart [1]. Internal organ involvement, especially cardiopulmonary involvement, can lead to premature death [2]. Evidence is accumulating that immunomodulation if applied early in the disease course could prevent organ dysfunction and improve prognosis. However, to start timely treatment in SSc, doctors are challenged to diagnose the disease in an early phase. Diagnostic criteria for SSc are lacking; however, classification and subtype criteria are available [3, 4]. Based on the extent of skin thickening, SSc is divided into two subtypes, namely limited cutaneous SSc (LcSSc) and diffuse cutaneous SSc (DcSSc) [1, 5]. To assess extent and degree of skin thickening, the modified Rodnan skin score (mRSS) is a commonly used, validated tool [6–8].

Systemic sclerosis starts with an early phase which is clinically characterized by Raynaud’s phenomenon and puffy fingers/hands. When specific autoantibodies and a typical scleroderma nailfold microscopic pattern are present, patients can be classified as early SSc according to the VEDOSS criteria [3]. The microvascular alterations like endothelial cell damage and increased vascular tone are already present in this early stage, probably caused by decreased production of vasodilators such as nitric oxide and increased production of vasoconstrictors such as endothelin 1 [9, 10]. This process combined with apoptosis of endothelial cells and subsequent loss of capillaries results in a decrease in blood flow and tissue hypoxia with a typical clinical manifestation known as Raynaud’s phenomenon [11, 12]. The blood vessel alterations can be visualized by nailfold microscopy often showing a specific SSc pattern [13–15]. Alterations in the nailfold microscopy pattern are however only indicative of lower oxygenation of the finger if the capillary density is less than 7 capillaries per millimeter. Furthermore, in the very early phase, not all SSc patients display an altered pattern. Since the structure and the function of the blood vessels can be altered early in the disease process and severe tissue hypoxia may be involved in disease progression, non-invasive techniques for the evaluation of the microcirculation and oxygenation are of importance to detect patients without nailfold alterations.

Optical imaging can be an excellent tool to monitor the blood vessel network and its oxygen saturation in real time and non-invasively. One of the optical techniques that gained interest among researchers and clinicians in recent years is photoacoustics, due to its capability of providing information about the blood vessel network at relevant depths with high resolution unlike other optical imaging techniques [16, 17]. Photoacoustic imaging is based on the thermoelastic effect, where absorption of short-pulsed light by endogenous chromophores, such as red blood cells, or exogenous chromophores, such as fluorophores, leads to instantaneous volume expansion of the red blood cells, resulting in generation of acoustic waves at megahertz frequencies [18]. These waves can be received by diagnostic ultrasound equipment and reconstructed to form an image of the adsorbed optical energy [19, 20]. By using multiple wavelengths in the near infra-red (NIR) therapeutic window, detailed discrimination between oxygenated and deoxygenated hemoglobin can be made and blood oxygen saturation can be derived from the measurements [21]. Photoacoustics has been utilized with success for a variety of biomedical applications, such as measurement of angiogenesis and blood oxygen saturation [22], detection of metastasis in melanoma patients [23], breast cancer [24], and synovitis in finger joint [25]. Recently, few studies have shown that photoacoustics can be used to look at skin microvasculature in the palm of the hand or the nailfold [26–28]. A small study with photoacoustics in SSc looked at microvascular dysfunction and disease activity, showing lower oxygenated hemoglobin and total hemoglobin in patients with progressive SSc compared to stable SSc patients and healthy controls [29, 30] .

Furthermore, photoacoustic can be combined with high-frequency ultrasound. High-frequency ultrasound, with a bandwidth of 13–24 MHz, is used to measure very shallow surfaces such as skin and has higher resolution than conventional ultrasound imaging. Adding this measurement to photoacoustic could lead to a more sensitive diagnostic tool of SSc because skin thickening is one of the hallmarks of the disease.

In this cross-sectional study, we explore the feasibility of the combined use of photoacoustic and high-frequency ultrasound in early SSc and SSc patients, individuals with primary Raynaud’s phenomenon, and healthy individuals to analyze the oxygen saturation of blood vessels in the nailbed. We use high-frequency ultrasound to determine skin thickening between the nailfold and the distal interphalangeal joint of the third finger in these different groups. We hypothesize that photoacoustic and high-frequency ultrasound can distinguish the early SSc and the SSc group from the individuals with primary Raynaud’s phenomenon in oxygen saturation and skin thickness. We also hypothesize that individuals with primary Raynaud’s phenomenon do not differ in oxygenation and skin thickness from healthy controls.

## Methods

### Design

This cross-sectional diagnostic pilot study was performed at the Department of Radiology and Nuclear Medicine and the Department of Rheumatic Diseases of the Radboud University Medical Center in Nijmegen, the Netherlands. The Department of Rheumatic Diseases is a tertiary referral center for patients with systemic sclerosis. Early SSc patients and SSc patients, individuals with primary Raynaud’s phenomenon, and healthy controls were asked to participate in the study. Measurements were done between October 2018 and December 2018. The study protocol was reviewed by the local ethics committee (No NL59142.091.16). All patients and volunteers received information and gave written informed consent prior to enrolment.

### Patients/subjects

Participating patients with SSc were classified according to the ACR-EUALR 2013 criteria [4]. All early SSc patients fulfilled the VEDOSS criteria [3, 5]. Baseline demographic clinical data were collected through chart review. These data included age, gender, disease phase (early SSc/SSc), mRSS, and medication use. Subjects with primary Raynaud’s phenomenon and healthy controls had no signs of underlying disease. Smoking and beta blockage use at moment of inclusion were exclusion criteria for all groups. Digital ulcers and/or finger contractures of the third finger on both sides were exclusion criteria only in the SSc patients.

### Assessments

All measurements were performed in a room with a fixed temperature of 22 °C. Patients stayed in the room 20 min prior to the measurements. The fingers of patients and subjects were examined with an optical probe videocapillaroscope equipped with a × 200 contact lens, followed by photoacoustic (PA) and high-frequency ultrasound (HFUS) measurements by trained assessors, KD for PA and HFUS and BK for videocapillaroscopy. The third finger is the most sensitive for SSc specific deviations in the pattern; therefore, we chose to image the third finger with no preference for the right or left hand [31].

A specific holder was designed to stabilize the third finger, and the probe was placed by hand on the fingernail in a longitudinal direction. We used a hybrid Visualsonics PA/HFUS system (Visualsonics, Inc.) equipped with a laser unit with wavelengths ranging from 680 to 970 nm (photograph of the system in supplementary file). The pulse energy was kept below the American National Standards Institute (ANSI) limits of maximum permissible exposure. The system provides a possibility to measure the oxygen saturation using dual laser wavelength technique 750/850 nm. For PA measurements, we used a 21-MHz central frequency transducer, while for the evaluation of the skin thickness, we used a central frequency transducer of 40 MHz. About 70 measurements of oxygenation were performed in 2 min; the average of these measurements was calculated. The thickening of the skin was estimated between the nailfold and the distal interphalangeal joint of the third finger, measuring the thickness between the air and the bone. All measurements together were executed in 30 min.

### Outcomes

Nailfold capillaroscopic images of every finger of the participants were evaluated by two trained assessors to classify the nailfold pattern as normal, atypical, early, active, or late SSc [13–15]. The capillary density was measured in the third finger of all patients. The nailfold capillaroscopy was performed in 30 min.

The PA measurements were performed in the nailbed of the third finger in the area beneath the nail and above the bone. The oxygen saturation measurements were extracted and analyzed for each participant.

### Statistical analysis

Descriptive statistics were provided as median and interquartile range (IQR) or numbers with percentages (%) where appropriate. Considering the small sample sizes, Wilcoxon rank sum test was used to examine differences in skin thickness and saturation between all SSc patients and volunteers with PR and between the subgroup of early SSc patients and volunteers with PR. *p* values of ≤ 0.05 were considered statistically significant.

The Spearman rank correlation was used to analyze an association between capillary density and oxygen saturation of the fingers.

## Results

Data of 5 early SSc patients, 12 SSc patients, 5 individuals with primary Raynaud’s phenomenon (PRP), and 9 healthy volunteers (HV) was analyzed. Table 1 summarizes the baseline characteristics.

**Table 1:** Baseline characteristics

	Early SSc (*n* = 5)	SSc (*n* = 12)	PR (*n* = 5)	HV (*n* = 9)
Age in years (median, IQR)	40 (32–58)	59 (46–62)	29 (25–44)	34 (26–52)
Female (%)	80%	42%	60%	33%
Disease duration in months (median, IQR)	12 (5–29)	88 (33–108)	–	–
mRSS (median, IQR)	–	5 (3–10)	–	–
ILD (%)	–	58%	–	–
Co-medication use (%)				
Calcium antagonist	40%	42%	–	–
Angiotensin 2 inhibitor	0%	17%	–	–
Endothelin antagonist	0%	8%	–	–
None	60%	33%	100%	100%

Disease duration from first non-Raynaud’s phenomenon

*Early SSc* early systemic sclerosis, *SSc* systemic sclerosis, *PR* primary Raynaud’s phenomenon, *HV* healthy volunteer, *mRSS* modified Rodnan skin score, *ILD* interstitial lung disease

**Fig. 1:**
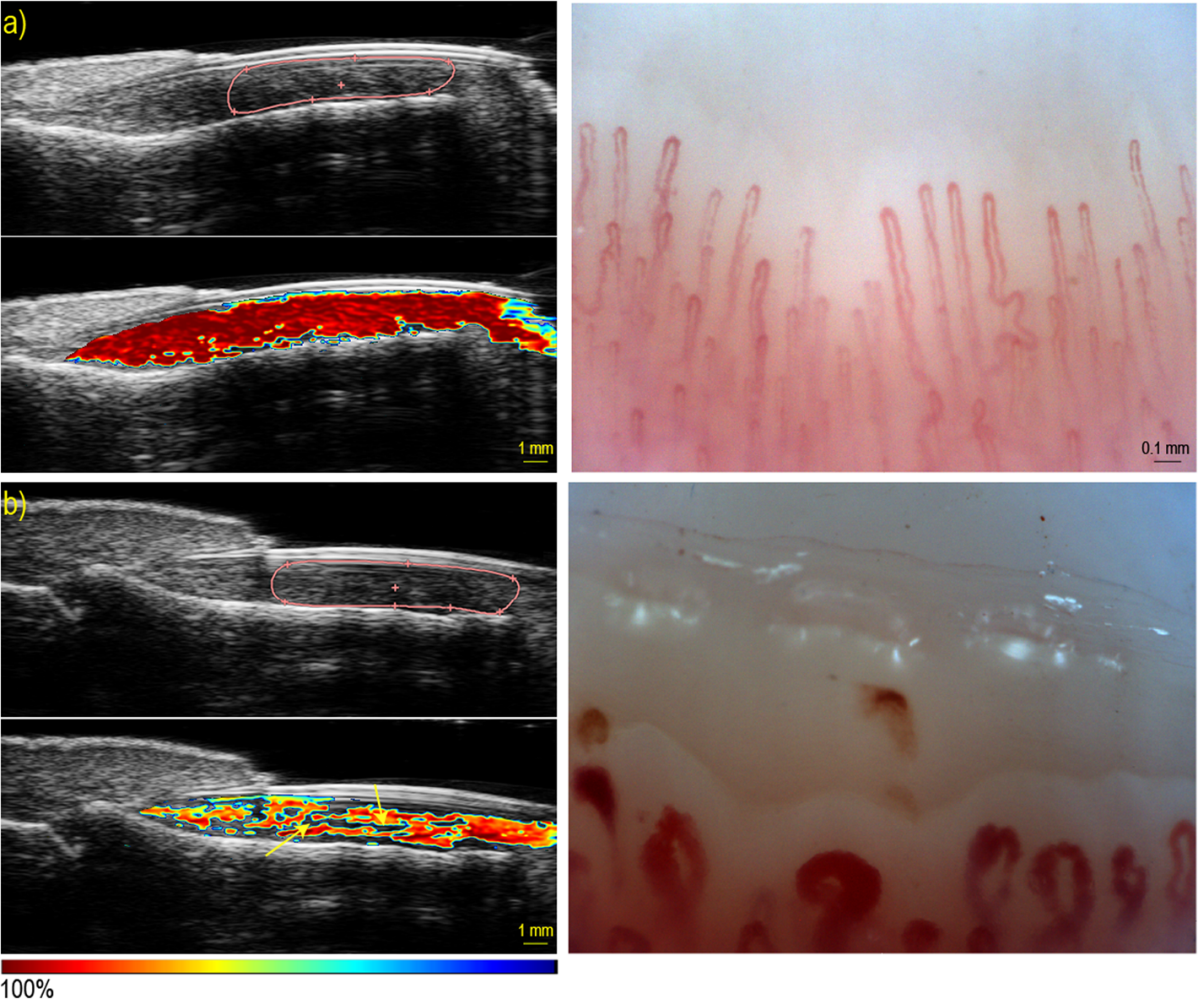
Photoacoustic and high-frequency ultrasound (left) and nailfold microscopy (right) images in a healthy volunteer (**a**) and an SSc patient (**b**)

Figure 1 shows the PA and HFUS images of a healthy volunteer and an SSc patient. The red line in the images delineates the region of interest for measuring oxygen saturation. The dark red color in the picture represents full oxygenation (100%). As shown in Fig. 1, there is a big difference in oxygenation between the healthy volunteer (a) where the area of interest is completely red and the SSc patient (b) where this area is fragmented light red. These findings indicate normal oxygenation in the healthy volunteer and a decreased oxygenation in the SSc patient. To calculate the degree of oxygenation in the area of interest, an average estimation of oxygen saturation over the region in 2 min is extracted. The dark regions are not taken into account when estimating the average oxygen saturation.

On the left is the ultrasound image where the red line delineates the region of interest where the oxygen saturation was estimated, and the white areas indicate a region with no oxygenation.

On the right side are the nailfold capillary microscopic images of a healthy volunteer with normal density and normal capillaries and below is an image of an active SSc pattern on nailfold microscopy with decreased density, giant capillaries, and hemorrhages.

### Oxygenation saturation

**Fig. 2:**
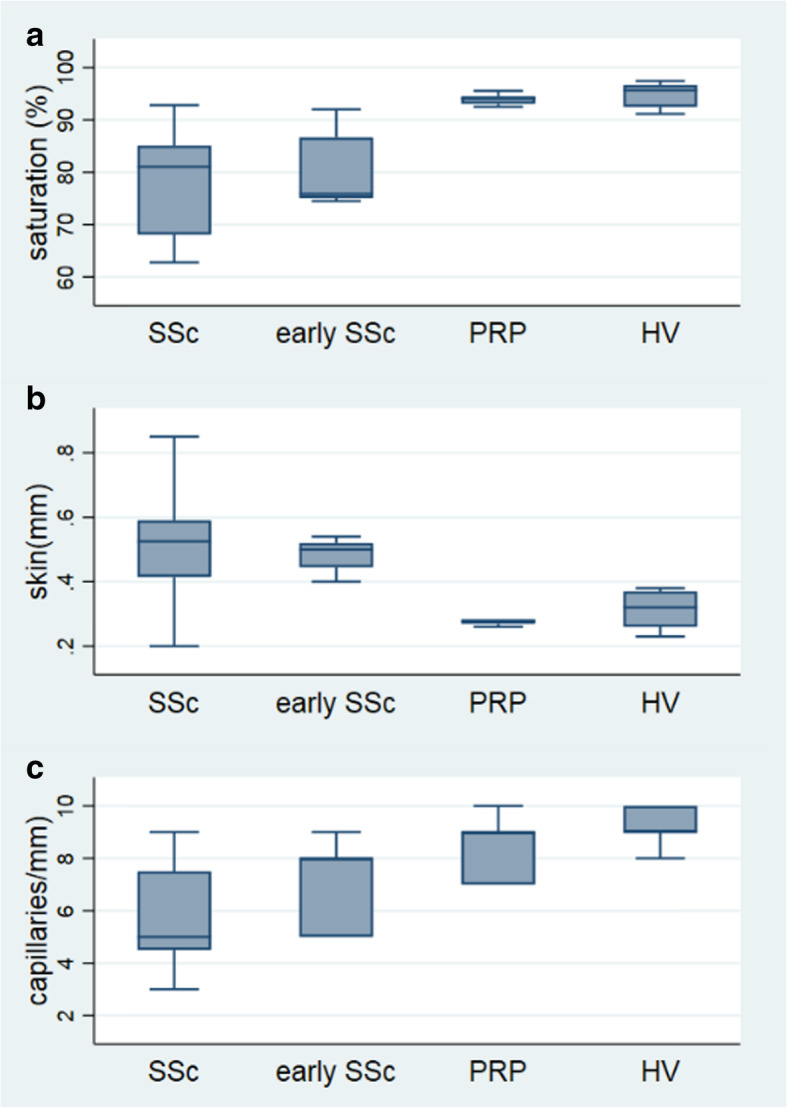
**a** Oxygen saturation (%), **b** skin thickness (mm) between the nailfold and the distal interphalangeal joint of the third finger, and **c** capillary density (capillaries per millimeter). SSc, systemic sclerosis; early SSc, early systemic sclerosis; PRP, primary Raynaud’s phenomenon; HV, healthy volunteer. Box plot between 25th and 75th percentile, line at median

There is a significant difference in oxygen saturation between the early SSc patients with the SSc patients and PRP individuals (*p* = 0.0002). The median oxygen saturation was 75.9% (IQR 75.1–86.6%) for early SSc, 81.0% (IQR 68.1–85.1%) for SSc, and 94.1% (IQR 93.1–94.5%) for PRP individuals, respectively (Fig. 2).

Comparing early SSc patients with PRP individuals, also a statistically significant difference in oxygen saturation (*p* = 0.0079) was observed. Figure 2 illustrates that oxygen saturation of PRP individuals is comparable with healthy volunteers.

### Skin thickening

**Fig. 3:**
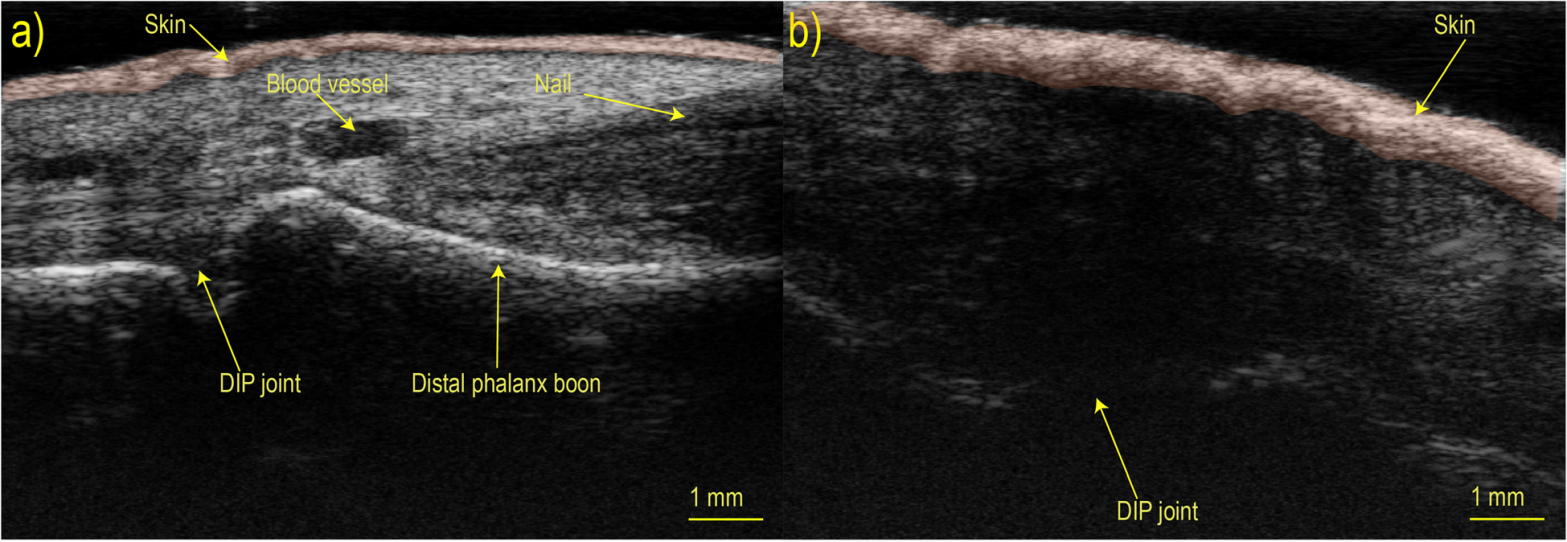
HFUS image of the skin thickening of a healthy volunteer and a SSc patient between the nailfold and the distal interphalangeal joint of the third finger. DIP joint, distal interphalangeal joint. High-frequency ultrasound image of the finger with a notation indicating different parts of the finger. The image **a** is for healthy volunteer and image **b** is SSc patient

Figure 3 shows a HFUS measurement of the skin between the nailfold and the distal interphalangeal joint of the third finger of a healthy volunteer and an SSc patient, and there is a clear difference in skin thickening and structure. The median skin thickness for the early SSc group was 0.50 mm (IQR 0.45 mm–0.52 mm), 0.53 mm (IQR 0.42 mm–0.59 mm) for the SSc group, and 0.28 mm (IQR 0.26 mm–0.27 mm) for the PRP group. We found a significant difference in skin thickness between the combined group of all SSc patients and PRP individuals and between early SSc patients versus PRP individuals (*p* = 0.0079).

**Fig. 4:**
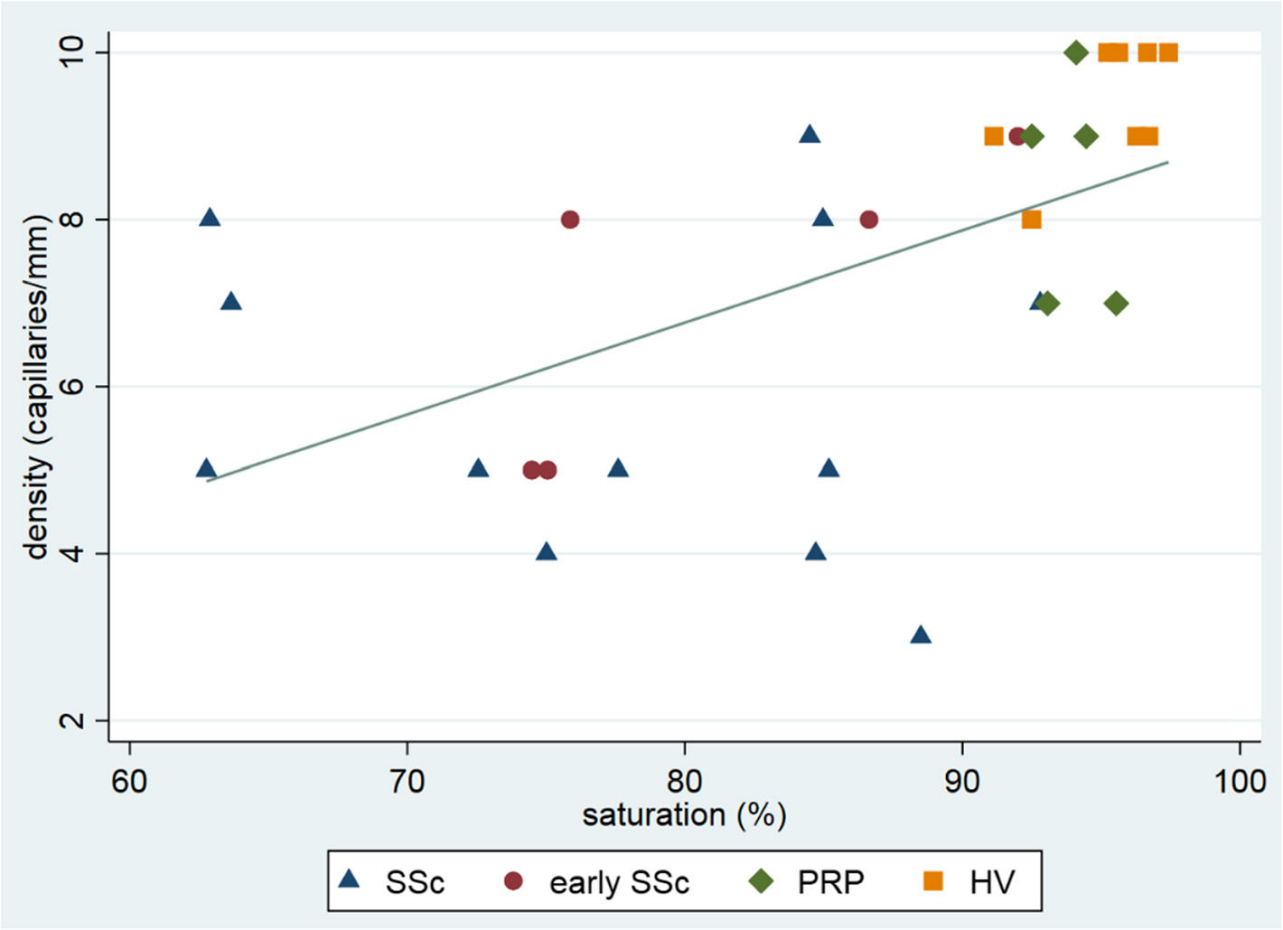
Scatterplot of correlation between oxygenation saturation and density. Oxygenations saturation (%). Density (capillaries per mm). Spearman’s rank correlation coefficient 0.68. SSc, systemic sclerosis; early SSc, early systemic sclerosis; PRP, primary Raynaud’s phenomenon; HV, healthy volunteer

In addition, we explored the association between capillary density and oxygen saturation. Figure 4 shows the scatterplots of the four groups. The spearman rank correlation coefficient was 0.68.

## Discussion

In this cross-sectional study, we investigated whether non-invasive measurements of oxygen saturation and skin thickening by two non-invasive techniques namely photoacoustic imaging and high-frequency ultrasound imaging could distinguish (early) SSc patients from individuals with primary Raynaud’s phenomenon. We found that oxygenation, measured by photoacoustic imaging, significantly differs between PR controls and both early SSc and SSc patients. Furthermore, we found a significant difference between PRP individuals and early SSc patients which is an important finding to help distinguish between primary Raynaud’s phenomenon and Raynaud’s phenomenon associated with early SSc. Using high-frequency ultrasound, we found a significant difference in skin thickening, measured between the nailfold and the distal interphalangeal joint of the third finger, between PRP individuals and (early) SSc patients. Combining photoacoustic and high-frequency ultrasound imaging as extra measurements to the diagnostics for patients with early SSc seems therefore promising.

There are limitations to the study. First, a small number of patients and controls were studied; our results need to be validated in a larger study population. Second, because photoacoustic and high-frequency ultrasound imaging is time-consuming with the prototype and the technic of positioning the finger we used, we were not able to perform measurements on all fingers. Examining all fingers could give a more detailed view of the overall oxygenation and skin thickening in all fingers. The risk of performing measurements in only one finger is missing deviations. However, recent developments in PA resulting in real-time imaging and assessment of saturation will allow measurements of all fingers in a much shorter time. Third, we excluded patients and controls using beta blockage and who were smoking, but there were differences in medicine use among patients. Most, but not all patients, used vasodilating medication which could lead to better oxygenation, including bosentan, an endothelin receptor antagonist, which was currently or previously used by some patients. Bosentan can potentially restore vascularization, and this could also lead to better oxygenation in those patients [32]. Fourth, we also did not correct for organ complications such as interstitial lung disease that may cause decreased oxygenation, which could also lead to a lower oxygenation measurement of the fingertips. None of the patients had pulmonary arterial hypertension, but almost 60% of the SSc patients had interstitial lung disease.

This study also has several strengths. It is the first study that shows the utilization of photoacoustics in combination with high-frequency ultrasound in early SSc. We were able to demonstrate the oxygenation difference between healthy and PRP individuals and (early) SSc patients. We were also able to assess skin thickening by high-frequency ultrasound as we found thickening of the skin between the nailfold and the distal interphalangeal joint of the third finger in (early) SSc patients but not in healthy or PR individuals. Even in early SSc patients, there are signs of skin thickening in the examined area compared to healthy and PR individuals. This skin thickening is not assessed by using the mRSS. Therefore, our results might contribute to early diagnosis of SSc but should be confirmed in a larger and prospective cohort of SSc patients including very early patients, fulfilling the VEDOSS criteria. In this follow-up study, it would also be interesting to look into the possible association of low oxygenation saturation and the development of digital ulcers. If this association exists, we could detect patients at risk for digital ulcers and start treatment more early to possibly prevent this disabling complication.

Follow-up studies should also focus on some other aspects of PA and HFUS. Currently, the measurements are time-consuming and should be optimized to make it more feasible for clinical practice. The focus should also be on what the predictive value of PA and HFUS in early diagnosis of SSc is compared to other diagnostics for early SSc, like nailfold microscopy. A large prospective study on patients with Raynaud’s phenomenon and a long follow-up time could help in answering these questions.

## Conclusion

In this pilot study, we demonstrated the possible significance of photoacoustics and high-frequency ultrasound to detect early SSc which needs further evaluation in a larger cohort. Both oxygenation and skin thickening were significantly different between early SSc patients and SSc patients and individuals with or without primary Raynaud’s phenomenon.

## Supplementary Information

**Additional file 1.**

## Data Availability

The datasets during and/or analyzed during the current study are available from the corresponding author on reasonable request.
