# Supplementary figures and images for: Photoacoustic and high-frequency ultrasound imaging of systemic sclerosis patients
Source: Arthritis Res Ther. 2021 Jan 12;23:22. doi: 10.1186/s13075-020-02400-y (PMC7802269; doi:10.1186/s13075-020-02400-y)

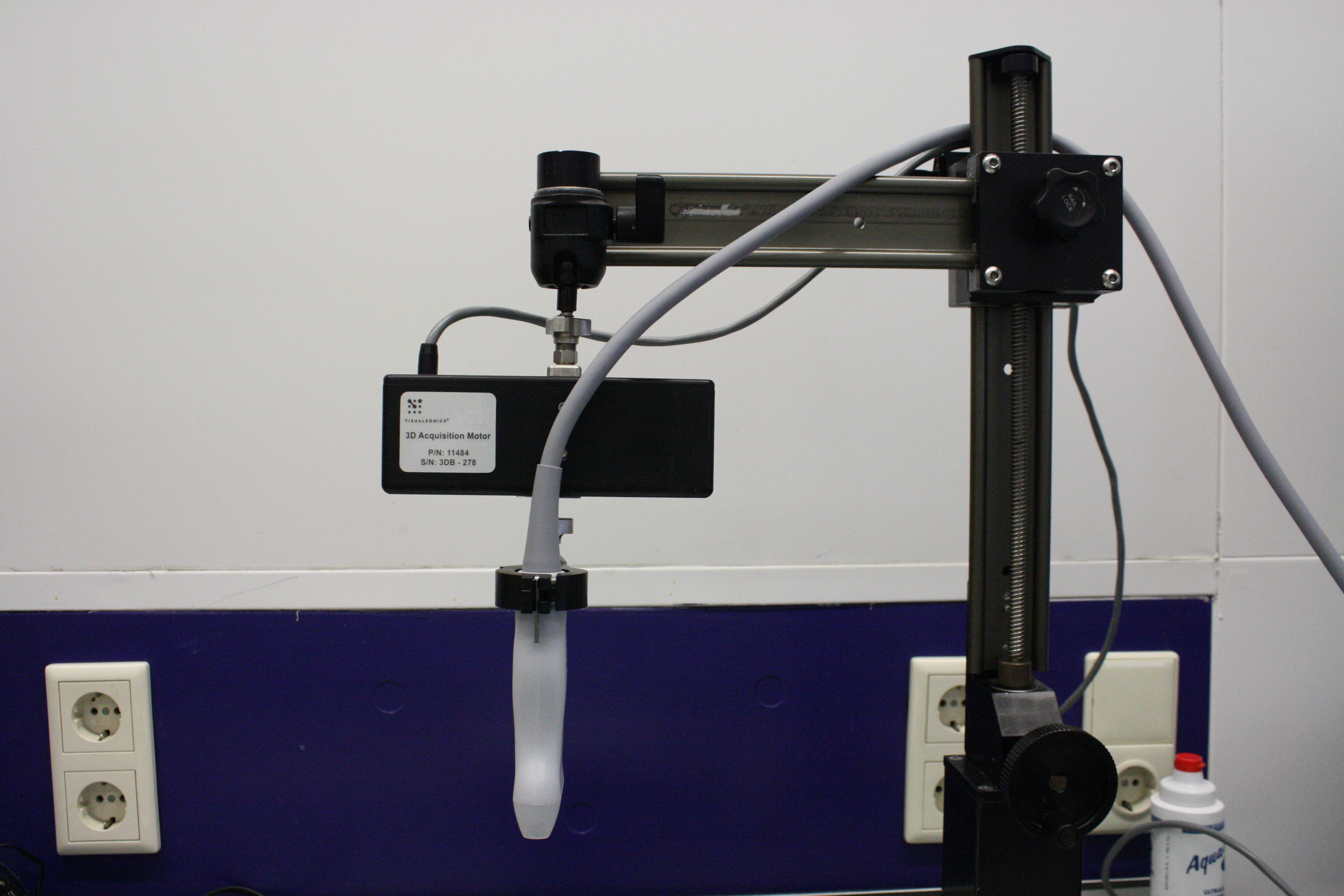

Supplement: Supplementary file 1 — Additional file 1. [file 13075_2020_2400_MOESM1_ESM.jpg]
